# *Lactobacillus plantarum* with Functional Properties: An Approach to Increase Safety and Shelf-Life of Fermented Foods

**DOI:** 10.1155/2018/9361614

**Published:** 2018-05-28

**Authors:** Sudhanshu S. Behera, Ramesh C. Ray, Nevijo Zdolec

**Affiliations:** ^1^Department of Fisheries and Animal Resources Development, Government of Odisha, Bhubaneswar, India; ^2^Centre for Food Biology Studies, 1071/17 Jagamohan Nagar, Khandagiri PO, Bhubaneswar 751 030, Odisha, India; ^3^Department of Hygiene, Technology and Food Safety, Faculty of Veterinary Medicine, University of Zagreb, Heinzelova 55, 10000 Zagreb, Croatia

## Abstract

*Lactobacillus plantarum* (widespread member of the genus* Lactobacillus*) is one of the most studied species extensively used in food industry as probiotic microorganism and/or microbial starter. The exploitation of* Lb. plantarum* strains with their long history in food fermentation forms an emerging field and design of added-value foods.* Lb. plantarum* strains were also used to produce new functional (traditional/novel) foods and beverages with improved nutritional and technological features.* Lb. plantarum* strains were identified from many traditional foods and characterized for their systematics and molecular taxonomy, enzyme systems (*α*-amylase, esterase, lipase, *α*-glucosidase, *β*-glucosidase, enolase, phosphoketolase, lactase dehydrogenase, etc.), and bioactive compounds (bacteriocin, dipeptides, and other preservative compounds). This review emphasizes that the* Lb. plantarum* strains with their probiotic properties can have great effects against harmful microflora (foodborne pathogens) to increase safety and shelf-life of fermented foods.

## 1. Introduction

Lactic acid bacteria (LAB) have been used for centuries for feed and food fermentation [1–3]. They are frequently taken up in the fermentation of vegetables, fruits, fish, meat, and milk [4, 5], improving texture and flavor of bread [6], sausages [7], and wine [8], suppress the microbe-dependent spoilage of food [9], and prolong the shelf-life [9]. Several microbial species of LAB establish themselves from mouth and gut to large intestine of human beings and thus serve as potential mucosal vaccines [10]. LAB is a diverse group of Gram-positive, anaerobic-aerotolerant homofermentative bacteria and L-(+)-lactic acid (LA) producer [11] and for* Lactobacillus* is perhaps the most predominant genus [10]. From the populous* Lactobacillus*,* Lb. plantarum* is the most versatile species/strain with useful properties and usually found in numerous fermented food products [12]. Moreover,* Lb. plantarum* is widely employed in industrial fermentation and processing of raw foods and “generally recognized as safe” (GRAS) and has qualified presumption of safety (QPS) status [2, 13].* Lb. plantarum* strains must have a high ability to survive in the gastrointestinal tract (GI) and adhere to its epithelial cells and most importantly be a safe strain (FAO and WHO) of animals and human [14]. “Fermentation” or “food being fermented” is the nonrespiratory metabolism of substrates (mainly organic compounds) on action of enzymes or microorganism so that desirable biochemical change results in significant refinement of the food [1, 2]. The importance of fermented foods is to increase the shelf-life of raw food matrices and also known to influence quality and functionality of foods by improving the taste and flavor fermented foods [3]. Positive perceptions of microbes are thus associated with desired changes in the food raw material during fermentation (fermented food) and beneficially impacting host health. Traditionally, fermented foods have been valued by many cultures for their health benefits and even therapeutic properties [3, 15]. Consumers worldwide are becoming increasingly aware of the relationship between fermented food and health, and the markets for so-called “functional foods” have been growing in recent years. Experts estimate that among functional foods probiotic foods comprise 60–70% of the total markets [16].

## 2. Systematics and Molecular Taxonomy

The species of the genera* Lactobacillus* are the dominant microbes found in human nutrition and in food microbiology, especially in fermented food systems [17].

*Scientific Classification*

 
*Domain*: Bacteria
 
*Phylum*: Firmicutes
 
*Class*: Bacilli
 
*Order*: Lactobacillales
 
*Family*: Lactobacillaceae
 
*Genus*:* Lactobacillus*
 
*Species*:* Lb. plantarum*

*Lb. plantarum*-group (LPG) comprises five closely/adjoining taxonomical species:* Lb. paraplantarum*,* Lb. pentosus*,* Lb. fabifermentans*,* Lb. xiangfangensis*, and* Lb. plantarum* (subsp.* plantarum* and subsp.* argentoratensis*) [26, 27]. Devi et al. [28] screened five distinct subspecies of* Lb. plantarum*-group (LPG) from fermented vegetable products keeping potential probiotic functionality.

### 2.1. Molecular Screening of* Lb. plantarum* Strain

The conventional methods of distinguishing/screening of* Lb. plantarum* strains depend on phenotypic tests (e.g., morphological and biochemical analyses) [27]. The conventional/traditional methods also involve a comparison of viable cell counts on agar plates and microbial turbidity measurements (600 or 620 nm) [35, 42]. Recently, next generation “omics-” methods aiming at a nonbiased and nontargeted detection of genes (genomics), mRNA (transcriptomics), protein (proteomics), metagenome (metagenomics), and metabolites (metabolomics) and diverse meta-analyses have been applied to investigate* Lb. plantarum *strains in more detail and on a systems biology level [45] (Table 1). Such expedites generate untold opportunities to expand our understanding of the role of* Lb. plantarum* involved in economically important fermentation [46]. The molecular based or “omics-” methods using polymerase chain reaction (PCR) and sequencing of the 16srRNA gene have been developed. These techniques are widely used for LAB* (Lb. plantarum)* identification and allow differentiation between strains of same species [24]. Pérez-Díaz et al. [46] studied the amplification (PCR) and analysis [nuclear magnetic resonance (NMR)] of the 16SrRNA gene of* Lb. plantarum* (dominant bacteria) present in fermented cucumber using AthoGen's proprietary technology and databases. The perception limit of the assay is found to be ~10^4^ CFU/mL. Genotyping (subtyping) methods can contribute information about the relativity of strains within a species. The random amplification of polymorphic DNA-PCR (RAPD-PCR) is an important genotyping technique commonly used for identification and determination of* Lb. plantarum *[27]. The RAPD process/technique is cost effective, is easy to execute, does not require prior sequence information, and needs only a little amount of template genomic DNA [27, 47]. Dong et al. [48] had developed a new genotyping method, to differentiate antifungal* Lb. plantarum* strains. The genotyping method used an alternative to RAPD method which is the multilocus variable number tandem repeats analysis (MLVA) from an array of sources (e.g., cheese, silage, sauerkraut, vegetable, and a probiotic product). The MLVA method is found to be better than RAPD-PCR method and provided valuable information for the application of biopreservative strains to reduce mold spoilage in food. More recently, pulsed-field gel electrophoresis (PFGE) has been widely used as a tool for the analysis of the genomic diversity of* Lb. plantarum* and also for identification and characterization of LAB from different food sources and geographical region to subspecies and strain level [24]. Some studies have been reported regarding the nutritional requirements for* Lb. plantarum* growth. Complete whole genome sequences of several* Lb. plantarum* strain such as* Lb. plantarum* WCFS1,* Lb. plantarum* ST-III, and* Lb. plantarum* P-8 have become available, and these sequences have revealed that* Lb. plantarum* bacteria are multiple amino acid and vitamin auxotrophs [49, 50]. Even if the genomic sequences of several* Lb. plantarum* strains are presently available, there are still limited reports on the function of genes from these bacteria [51].

### 2.2. Genome and Genome size of* Lb. plantarum* Strain

As a result of a process called “genome reduction”* Lb. plantarum* strains have relatively small genomes, varying from 1.8 to 3.3 Mbp [45]. Liu et al. [49] organized genome sequence and comparative genome analysis of* Lb. plantarum* (strain 5-2), obtained from fermented foods from Yunnan province, China. The strain was resolved to consist of 14 insertion sequence (IS) elements (3114 genes). There were DNA replication proteins (24 nos) and DNA repair proteins (76 nos) in 5-2 genome which encodes vital enzymes needed for phosphoketolase (PK) and Embden-Meyerhof-Parnas (EMP) pathways. However,* Lb. plantarum* LL441 (isolated from cheese) contain 29 open reading frame (ORFs) encoding glucosidases belonging to different hydrolase families [52].

## 3. *Lb. plantarum* in Traditional Food Systems

The market for fermented food and ingredients has been growing in recent years and is expected to grow from $636.89 billion in the year 2016 to $888.76 by 2023 [53]. The fermented food is induced by consumer demand for food products that are virginal, healthy, fresh-like, minimally processed, and nutritious [54]. To preserve the essence/attribute of the particular fermented food products, techniques are needed to retain organoleptic properties and to promise an acceptable shelf-life [53]. Hitherto, fermented dairy product (e.g., yogurt) has been designed both as a potential source of beneficial (probiotic) strains and as a standard form/matrix for offering such functional strains. Nevertheless, part of the mainstream now shifted to a field of nondairy fermented vegetables and fruits of Asia and fermented plant raw material, in particular cereal, of Europe and Africa as ecosystem of potentially beneficial strains [3, 55]. However,* Lb. plantarum* is a potential probiotic and is mainly from fermented food systems [49], including* pickles*,* sauerkraut*, Korean* kimchi*, brined* olives*,* sourdough*, Nigerian* Ogi*, and other fermented fruits and vegetables and also some* cheeses*, fermented* sausages*, and* stockfish* (unsalted fish, especially cod) [49, 56] (Table 2).

### 3.1. Fermented Vegetable Products

Among the several species identified in fermented vegetables,* Lb. plantarum* accounts for lion's share in fermented vegetables due to its ability to resist high saline and acidity content of fermented vegetables, mainly cucumber,* sauerkraut*, and olive [56]. Moreover, strains of* Lb. plantarum* have been treated as acceptable starter cultures giving an array of fermented vegetable products [57, 58]. Several studies reported resulting lactic-fermented food products from sweet potato (*Ipomoea batatas* L.), that is, lactopickles [58–60], curd [61, 62], and lactojuice [57, 59, 60], employing* Lb. plantarum* (MTCC 1407) as the starter culture. In a study, Panda and Ray [57] reported that sweet potato (fully boiled/nonboiled) was pickled (LA fermentation) by preprocessing, cut, and blanched in brine solution (NaCl, 2–10% w/v) using a probiotic strain of* Lb. plantarum* (MTCC 1407). The developed pickled sweet potato [pH of 2.9–3.0, LA of 2.6–3.2 g/kg, titratable acidity of 2.9–3.7 g/kg, and starch of 58–68 g/kg (on fresh weight basis)] was found acceptable by consumers. More recently, Behera et al. (2018) optimized the process parameters (e.g., inoculums volume, salt concentration, and incubation period) for pickling of elephant foot yam* (Amorphophallus paeoniifolius)*. The results claimed that the 8% (w/v) of NaCl concentration, 10% (v/v) of inoculums volume, and 22 d of incubation period were found effective for maximum yield of LA.

*Kimchi* is an accustomed Korean fermented food formed from Chinese cabbage.* Kimchi* contains various LAB strains, including* Leuconostoc* sp.,* Lactococcus* sp.,* Lactobacillus* sp., and* Weissella *sp. The LAB strains that are present during* kimchi* fermentation are believed to have possible probiotic properties and health benefits [63]. Son et al. [34] investigated the possibility of using* Lb. plantarum* Ln4, one of several strains isolated from* kimchi*, as a probiotic according to its characteristics as compared to commercial probiotic and yogurt starter strains.* Lb. plantarum* wikim 18 (KFCC 11588P) isolated from* baechu* (napa cabbage)* kimchi* exhibited probiotic trait [42]. The strains of LAB (*Lb. plantarum* B282) were successfully employed as starter in green olive (Spanish-style) fermentation [64].* Lb. plantarum* was the first LAB associated with cucumber fermentation [46]. Pérez-Díaz et al. [46] conducted cucumber fermentation by using* Lb. plantarum* (2 × 10^8^ CFU/mL), primarily* Lb. plantarum* found in brine and able to produce 0.6–1.2% lactic acid [46]. Abadi Sherahi et al. [65] studied the effect of* Lb. plantarum* ATCC 8014 fermentation on oxidative stability effects of olive oil. The fermented olive fruits were suggested as an appropriate method for preservation of olive quality and olive oil stability during storage.* Sa Taw Dong* is a traditional fermented sticky bean product in the Southern Thailand. Saelim et al. [66] isolated functional properties of* Lb. plantarum* S0/7 from a fermented stinky bean and showed potential probiotic properties.

*Lb. plantarum *was also isolated from starchy wastes. The cassava starch is a potential source of LA. In a study, Bomrungnok et al. [67] claimed that lactic acid (LA) produced from cassava starch using high dilution rate and cell density of* Lb. plantarum* (SW14) in a continuous mode of operation.* Fufu* is a fermented wet paste obtained from cassava starch, regularly eaten up in many parts of West Africa. Species of LAB fitting for the genera* Leuconostoc, Streptococcus*, and* Lactobacillus* are the dominating microorganisms in* fufu*. The spontaneous food fermentation has several disadvantages (e.g., survival of food pathogens, spoilage of fermented products, and long duration), which are still prevailing at the household levels in Africa. Thus, the use of starter cultures is favored as active/speedy acidification of the finished products and also lowers the pH to a certain point, which can inhibit the growth of undesirable/unpalatable bacteria [31]. Rosales-Soto et al. [31] studied fermentation of fortified cassava flour (with protein and provitamin A) using* Lb. plantarum* (strain 6710) as starter. The fermentation of fortified cassava flour resulted in production of wet* fufu* which is well accepted by consumers.

### 3.2. Fermented Cereal Products

Fermented cereal commodities are critical/valuable sources (e.g., proteins, carbohydrates, minerals, vitamins, and fiber) of nutrition [68]. The nonalcoholic and alcoholic food commodities (e.g.,* Mageu*,* Togwa*,* Gowe*,* Poto-Poto*, and* Degue* and* Obushera*) are obtained from cereals (e.g., rice, wheat, maize, sorghum, and millet) and are voluminously accepted in several regions of the world. LAB are the prevalent microorganisms (e.g.,* Leuconostoc*,* Pediococcus*, and* Lactobacillus*) associated with the fermentation of cereal-based foods and beverages; however, yeasts are also habitually reported but at lower orders of magnitude [69]. A traditional Turkish cereal-based (prepared from wheat flour) and LA fermented food product called* “tarhana”* is mostly produced at home or home-scale level. The fermented finish product* (tarhana)* is a sequel action of mixed population of microbes (e.g.,* Lb. plantarum* and* Lb. brevis*) [70].* Ogi* is a traditional fermented product usually obtained from spontaneous and uncontrolled fermentation of cereals (e.g., maize, sorghum, or millet). Several groups, including LAB, yeasts, and molds are deliberately involved in* Ogi* fermentation, although* Lb. plantarum* is the dominant one [71]. Traditional fermented beverage of* “bushera”* (prepared from sorghum and millet flour) is widely consumed in Uganda [71]. The LAB from household* bushera* included* Lb. fermentum, Lb. brevis*,* Streptococcus thermophilus, *and* Lb. plantarum *[72].* Boza* (prepared from maize, millet, wheat, rye or rice, and other cereals) is a historic fermented beverage used up in the countries of Balkan province, including Albania, Bulgaria, Romania, and Turkey [73]. Several LAB species (e.g.,* Leuconostoc mesenteroides*,* Lb. fermentum, Lb. pentosus, Lb. rhamnosus, *and* Lb. plantarum*) screened from* boza* provide antimicrobials (bacteriocins), increasing the shelf-life of the finished product and manifesting health benefits [74, 75]. Since 1994, an oats-based probiotic beverage known to be the first commercial product is called* “proviva*.” The addition of probiotics (*Lb. plantarum* 299v) and a liquefying agent (malted barley) added beneficial effects to consumers [76]. Another nonalcoholic cooked beverage, named* “uji”* (prepared from sorghum, maize, or finger millet), fermented with LAB (especially* Lb. plantarum*) [76].* Togwa* LA fermented product is made from either cereals (maize, millet, and sorghum), root tuber of cassava, or their combinations [77]. Microbial communities of* Togwa* are diverse and comprise LAB of the genera* Lactobacillus* (*Lb. brevis, Lb. cellobiosus, Lb. fermentum, *and* Lb. plantarum*) [78]. Nyanzi and Jooste [69] reported a symbiotic functional drink from the oats by participating a probiotic culture (*Lb. plantarum* A28) for the production of fermented beverages.* Pozol* is a maize based probiotic beverage consumed in the Southeastern Meico [77]. Recent studies showed that LAB (e.g.,* Lb. plantarum*) can have a great impact on fabricating the* pozol *microbial community [77].

### 3.3. Fermented Meat, Fish, and Dairy Products

Lactic acid bacteria (LAB) are involved in fermentation of many different kinds of animal foodstuffs such as meat, fish, or dairy products. Meat fermentation involves natural LAB or added starter cultures. Specific spontaneously naturally fermented sausages are developed by activity of well adapted strains in meat and environmental conditions, usually called “house microbiota” [79]. In that sense,* Lactobacillus plantarum *strains showed a high diversity in specific dry fermented sausages, even in the same product of different producers [79, 80]. This species has been shown as dominant one in many traditionally fermented sausages worldwide, that is, in Mediterranean countries [80–85], Asia [86–88], South America [80], or Africa [89]. Characterization of dominant* Lb. plantarum *from fermented sausages is revealed in many strains proposed as potential functional starter cultures with desirable technological and safety properties [23, 37, 89, 90]. One of the most studied properties of indigenous* Lb. plantarum *strains is ability to produce bacteriocins* plantaricins*, antimicrobial peptides usable in different food matrices for reduction of sensitive bacteria including foodborne pathogens or spoilage bacteria [91]. Starter cultures or protective cultures have been widely used within the strategies of improving safety and quality of fermented sausages [92–94]. In this respect, implementation of selected autochthonous or commercial* Lactobacillus plantarum *cultures in sausage production contributed to reduction of biological hazards such as pathogens [95] or biogenic amines [96] as well as enhancing sensorial properties of final products [90]. Even with controversial concept in meat industry, the probiotic strains of* Lb. plantarum*, both novel and commercial, are intensively studied in fermented sausages in order to develop the so-called probiotic sausages [97–99].

*Lactobacillus plantarum *strains with technological and food safety properties are also commonly found in traditional fish products. For example, Zeng et al. [100] selected strains from “Suan yu” with good acidification rates, antimicrobial activities, moderate proteolytic activity, and low (if any) amino acid decarboxylase activity. When implemented to fermented surimi,* Lb. plantarum *cultures presented favorable technological properties revealed in high overall acceptability of product. From the point of view of product safety,* Lb. plantarum *culture applied to fish fermented sausage significantly reduced the formation of biogenic amines putrescine and cadaverine during fermentation [101].

Considering dairy products, Quigley et al. [102] emphasize the rare finding of* Lb. plantarum *in raw milk and its low technological importance in standard milk processing. However, strains with probiotic properties have been isolated from different dairy-related niches, for example, camel milk [25], cow's or ewe's raw-milk cheeses, and whey [103–106]. Hence, recent studies are more focused on implementing probiotic strains of* Lb. plantarum *in fermented milk beverages or cheeses to gain novel products with enhanced health benefits [107, 108].

### 3.4. Ethnic Fermented Food

Fermented bamboo shoots or smoked and salted fish or local traditional fermented food is categorized under ethnic fermented foods. Fermented bamboo shoots (e.g.,* Eup*,* Ekung*,* Hecche*,* Hirring*,* Soidon*, and* Soibum*) are nonsalted acidic products obtained by fermentation with LAB, especially by* Lb. plantarum* (range of 10^8^ CFU/g). LAB* (Lb. plantarum)* is the dominant microorganism in ethic fermented foods like* idli*,* dosa*, and* dahi,* which are made locally in India and other South Asian countries (e.g., Pakistan and Bangladesh) [55, 109]. In a study, Catte et al. [110] reported that the strains of* Lb. plantarum *are often screened from seafood products, predominately from salted and smoked fish products.* Lb. plantarum* is the most prolific LAB isolated from traditional cassava based fermented foods (e.g.,* agbelima*,* gari*,* fufu*, and* lafun*) from African countries [111].

### 3.5. Novel Foods, Mainly Plant Based Foods, and Beverages

The organic matter and starch content of cassava fibrous residues in semisolid fermentation produce LA using* Lb. plantarum* as starter culture. In a study, Ray et al. [109] investigated that high starch content (60–65% of dry weight basis) of cassava residues can convert to a maximum level of LA (about 63.3%) using* Lb. plantarum* MTCC 1407 as starter culture. Alcoholic fermentation is an essential step in producing high-quality vinegar and typically involves the use of pure yeast to initiate fermentation. Chen et al. [112] studied using mixed cultures of* Saccharomyces cerevisiae* and* Lb. plantarum* for preparation of citrus vinegar. The mixed culture in alcoholic fermentation found the flavor and quality of citrus vinegar effectively improved, indicating additional economic benefits of fermentation. However, Liu et al. [113] studied the investigation of the capability of* Lb. plantarum* BM-LP14723 to enter and recover from the viable but nonculturable (VBNC) state and to cause beer spoilage. The VBNC* Lb. plantarum* BM-LP14723 retained spoilage capability. The study presented that beer spoilage by* Lb. plantarum* can hide both in breweries and during transporting and marketing process and thus lead to beer spoilage incidents. In wine making, malolactic pathway of fermentation is generally followed in fermentable LAB species. However,* Lb. plantarum* is one of the species most widely used for malolactic fermentation in wine making [8]. The advantage of Lb. plantarum strain is the ability to grow/cope in the adverse wine environment, giving diverse and distinct metabolites/compounds that can improve organoleptic properties of wine. For this reason, the selective strain* (Lb. plantarum)* is essential for optimization and preservation during wine making [114]. Sweet lemon juice (rich source of vitamin C and essential minerals) was fermented with* Lb. plantarum* LS5 to produce a probiotic juice. The cell counts (*Lb. plantarum* LS5) increased from 7.0 to 8.63 log CFU/mL during fermentation (37°C for 48 h) and decreased from 8.63 to 7, 14 log CFU/mL after storage (4°C for 28 d) [115, 116].* Lb. plantarum*, incorporated in osmotically dehydrated apple cubes, survived over a storage period of 6 days at 4°C maintaining constant values of 10^7^ CFU/g in the apple cubes [53]. Freire et al. [16] developed a nondairy beverage based on the Brazilian indigenous beverage,* cauim*, by selecting a potential probiotic LAB strain (*Lb. plantarum *CCMA 0743) isolated from different Brazilian indigenous foods (*cauim*,* calugi*,* cairi*,* yakupa*, and* chicha*) to be used as starter culture in a blend of cassava and rice to increase the product's functional properties. Zhang et al. [44] investigated the impact of several baking conditions/factors (e.g., dough weight, temperature) and storage period on survival rate of* Lb. plantarum* (P8). Bread samples with varying dough weight (5–60 g) were baked at different temperatures (175–235°C) for 8 min and the residual viability of bacterial counts was determined every 2 min. The baking process significantly decreased the viability from 10^9^ CFU/g to 10^4-5^ CFU/g in bread, which contributes to the development of probiotic bakery products.

### 3.6. Metabolomics of* Lb. plantarum* in Fermented Foods

Food metabolomics or “foodomics” has been practiced and adopted in the study of different foods/fermented food products in the literature [117]. Specifically, in fermented food systems, it is practically used to estimate and monitor the changes occurring during the fermentation process and also to investigate the composition of fermented foods [118]. At the beginning, the composite/heterogenous components in fermented food need to be separated/simplified prior to detection. Several biochemical techniques are adopted by different authors for identification/detection of compositional mixture found in fermented food products. The biochemical technique such as gas chromatography (GC) alone or in combination with mass spectroscopy (MS) called “GC-MS” is one of the best used techniques for foodomics detection.

High performance liquid chromatography (HPLC) is a substitute tactics/method for GC-MS commonly used for metabolic analysis of fermented food products; however, in comparison to GC, HPLC has an inferior chromatographic resolution. Furthermore, HPLC is more convenient than other techniques and measures a wider range of analytes/components with higher sensitivity. Nuclear magnetic resonance (NMR) spectroscopy is another advanced analytical technique which is used in separation and recovery of spent analytes. Additionally, in NMR technique, diverse group of components can be measured by its high simplicity and reproducibility nature [119].

## 4. Quality/Functional Properties of Fermented Foods

### 4.1. Enzyme Systems

In spite of the ancient uses of LAB for production of fermented foods, their multipotential for enzyme production has recently generated much research interest. Therefore, enzymes from microorganisms have found a broad spectrum of industrial applications in the starch, beverages, food, and textile industries [111, 120].

#### 4.1.1. *α*-Amylase

*α*-Amylase (or 1,4-*α*-D-glucan glucanohydrolase) (EC 3.2.1.1) catalyzes the hydrolysis (cleavage of *α*-1,4 linkage) of starch (raw and soluble), while consequently liberating smaller dextrins and oligosaccharides. *α*-Amylase has been classified into the glycoside hydrolase family based on amino acid sequence classifications widely found among a wide range/diversity of microorganisms (e.g., actinomycetes, bacteria, molds, and yeasts) [121]. LAB (*Lb. plantarum* strains) are dominant microbiota involved in the fermentation of numerous carbohydrate-based foods [122]. Ray et al. [109] studied *α*-amylase production in submerged fermentation and optimized (response surface methodology) the process parameters (pH, incubation period, and temperature) using* Lb. plantarum* MTCC 1407 as a starter culture. Of particular importance, amylolytic LAB were found useful in modifying the structure and properties of starches for production of lactic acid and *α*-amylases extensively to improve bread making [6]. Amapu et al. [120] isolated* Lb. plantarum* (AMZ5) from maize flour that retains excelling starch degradation ability through production of reducing sugar yield and high extracellular amylase. Kanpiengjai et al. [121] identified the effect of starch binding domains (SMDs) on biochemical and catalytic properties of *α*-amylase obtained from* Lb. plantarum *S21. The results indicated that the C-terminal SBDs of* Lb. plantarum *S21 *α*-amylase showed substrate preference and substrate affinity and also the catalytic efficiency of the *α*-amylase without any changes in the degradation mechanisms of the enzyme.

#### 4.1.2. Esterase

*Lb. plantarum* are sources of a large variety of microbial ester hydrolases because they can produce a wide range of phenolic alcohols, short-chain esters, and fatty acids [51]. Kim et al. [51] identified and characterized a novel SGNH-type esterase (LpSGNH1) from* Lb. plantarum* WCFS1, immobilized for biotechnological applications, especially used for a potentially broad range of applications in food.

#### 4.1.3. Lipase

Microbial lipases (triacylglycerol acylhydrolases) (EC 3.1.1.3) owing to their broad substrate specificity are widely used in various industrial applications like food processing, organic synthesis, detergent formulation, and oil manufacturing [123]. Uppada et al. [123] used* Lb. plantarum *MTCC 4451 as a source of lipase for the purposes of ester synthesis and meat degradation. Andersen et al. [124] purified lipase from* Lb. plantarum* MF32, originally isolated from fermented meat. The apparent molecular weight of lipase was estimated to be approximately 75 kDa with isoelectric focusing (pl) value of 7.5 and 7.0. This enzyme has been shown to contribute to sensory quality and reduced production of fermented sausage.

#### 4.1.4. *α*-Glucosidase

*α*-Glucosidases of* Lactobacillus* have been little studied compared to other glycosidases [52]. Delgado et al. [52] isolated a strain,* Lb. plantarum* LL441, from the microbiota of a conventional starter free cheese made from milk. The ORFs of gene contain *α*-glucosidases.

#### 4.1.5. *β*-Glucosidase

*β*-Glucosidase (EC 3.2.1.21) catalyzes the hydrolysis/breakdown of aryl and alkyl *β*-glucosides (e.g., diglucosides and oligoglucosides) [125]. Several strains of* Lb. plantarum* showed *β*-glucosidase activity (Behera and Ray, 2017). Lei et al. [126] investigated that strains of* Lb. plantarum* (LP1, LP2, LP3, LP5, LP6, LP7, and LP11) showed *β*-glucosidase activity that degrades cyanogenic glycosides during spontaneous cassava fermentation. Gouripur and Kaliwal [127] had undertaken a study to isolate, screen, and optimize intracellular *β*-glucosidase production by* Lb. plantarum* strain LSP-24 from colostrum milk.

#### 4.1.6. Phosphoketolase-2 (EC 4.1.2.22)

Phosphoketolase-2 from* Lb. plantarum* accepts either xylulose-5-phosphate (Xu5P) or fructose-6-phosphate as substrate and plays an important role in energy metabolism [128].

#### 4.1.7. Enolase

The experimental data reported that the* Lb. plantarum* LM3 has the potential of binding human plasminogen (Plg). This work also provided the evidence that the cell wall fraction of* Lactobacillus* strain (LM3) surface-displayed enolase which has the capacity to bind to plasminogen [129]. Vastano et al. [129] analyzed* Lb. plantarum* LM3 for tolerance to GI environmental conditions, found intrinsically resistant to stimulate pancreatic juice and to bile salts.

#### 4.1.8. Lactate Dehydrogenase (LDH)

Lactate dehydrogenase (LDH) (EC 1.1.1.27) catalyzes the reduction/deduction of pyruvate to lactate, the major/dominant finished product in homolactate fermentation. Krishnan et al. [130] reported that whole cell of* Lb. plantarum* (NCIM 2084) revealed low levels of LDH activity, but cells treated with organic solvents like chloroform, diethyl ether, and toluene increased the activity of LDH.

### 4.2. Dipeptides/Bioactive Peptides

*Lb. plantarum* is well recognized, particularly because it can produce antimicrobial cyclic dipeptides (CDPs). Kwak et al. [36] reported the verified set of CDPs with antimicrobial activity from* Lb. plantarum* LBP-K10 against multidrug-resistant bacteria, pathogenic fungi, and influenza A virus. The result exhibited considerably higher antimicrobial activity against the tested pathogenic microorganisms.

### 4.3. Vitamins

Fermentation with food grade LAB is a good strategy to improve the nutritional values and vitamin contents of food products [131–133]. The addition of vitamins (folate, riboflavin, vitamin B12, etc.) producing* Lb. plantarum* into fermented milk, yogurt, or soybean could potentially increase the vitamin concentrations and supply nutrients to consumers [132]. The* Lb. plantarum* is more adjustable in the harsh condition of fermentation processes. The adaptability of* Lb. plantarum* to a fermentation process, their metabolic flexibility, and biosynthesis ability are some of the critical attributes that assist the progress and application of different strains of* Lb. plantarum* in fermented foods for in situ releasing, producing and/or increasing specific beneficial compounds (e.g., vitamin B2) [134]. Li et al. [132] studied isolating extracellular vitamin B12 (cobalamin) producing* Lb. plantarum* strains (LZ95 and CY2) from lab stocks and evaluated their probiotic potential for application in the food industry. Vitamin B12 producing* Lactobacillus* strains (60–98 *μ*g/L) was found to have good viability in bile salts (0.3%) and gastric acid (pH 2.0 and 3.0) as well as good attachment/adhesion to Caco-2 cells.

### 4.4. Development of Aroma, Flavor, and Texture in Fermented Food/Products

*Lb. plantarum* has an outstanding effect on the flavor and texture in fermented foods [24]. The quality of fermented food/products (e.g.,* kimchi*,* sauerkraut*,* jeotgal*, and* pickles*) could be improved by several* Lb. plantarum* strains, in terms of stability of quality, enhanced taste, and health-promoting benefits [135]. The microbial spoilage/decay of fruit juices has been implicated to mold, yeast, and acetic and lactic acid bacteria. The LAB* (Lb. plantarum)* develops an unsavory/undesirable buttermilk as a result of diacetyl and fermented flavor due to organic acid (LA, acetic acid) production. However, these* Lb. plantarum* strains also caused the swelling of packages due to the formation of carbon dioxide (CO_2_) [136]. Berbegal et al. [137] reported methodological characterization of* Lb. plantarum* strains isolated from Apulian/grape wines. Various factors (e.g., pH, ethanol tolerance, sugar, resistance to lyophilization, and presence of starter cultures) in grape wine were evaluated. However, coinoculation of* S. cerevisiae* and* Lb. plantarum* in grape improves the bacterial adjustment to harsh conditions of wine and shortened the fermentation time.* Lb. plantarum* is able to conduct the beer fermentation, which had antibacterial effects. Moreover, the occupancy of alcohol or related compounds, nearly low pH, and inadequate amounts of nutrients and oxygen results in high microbiological stability of beer [138]. However, in spite of the fact that these adverse conditions prevail, there is still possibility of developing of spoilage microorganisms that is manifested in turbidity increase and unpleasant flavor. Furthermore, some beer spoilage microorganisms are also able to produce pathogenic chemicals [113].

### 4.5. Biopreservative Compounds

The control/growth of one organism (undesired) by another has received much attention in recent years [139]. In fermented food series, biopreservation refers to the use/benefit of antagonistic microorganisms or their metabolic products to inhibit (or destroy) undesired microorganisms in fermented food products, thereby upgrading food safety and extending the shelf-life of foods [9]. Several methods of food and feed processing and preservation have been used to prevent/control the development of microorganisms in foods and consequently avoid the formation of toxins. Physical methods such as drying and irradiation as well as chemical preservatives, such as organic acids (e.g., sorbic, propionic, and benzoic acid), are most frequently used in food preservation. Further, consumers' demand for healthier and safer foods creates a need for use of natural solutions and derives researchers to investigate biological methods for the control of foodborne pathogens [139].

### 4.6. Exopolysaccharide (EPS)

EPS are the “food grade biopolymers” or high molecular weight extracellular biopolymers obtained from natural sources that are formed during the metabolic process of microorganisms, (e.g., bacteria, fungi, and blue-green algae) [140, 141]. Among the wide variety of EPS-producing microorganisms, LAB is generally regarded as safe because of the long history of secure applicability in substances for human utilization/consumption [142]. In addition, EPS has been reported to contribute the rheology of the fermented food and provides potential health-promoting properties in the advances of functional foods [141]. In the last decade, a large number of EPS-generating LAB have been isolated from a variety of fermented food systems (e.g.,* cheese*,* kefir*,* sausages*,* wine*, and* yogurt*). The species of LAB, such as those of* Streptococcus*,* Lactobacillus*,* Pediococcus*,* Lactococcus*, and* Bifidobacterium*, are frequently reported as EPS-generating microorganisms. However,* Lb. plantarum* is an eminent microorganism for its potential EPS-producing properties and received considerable attention [20]. It has been proved that several influencing factors depend on the yield of EPS. The composition of monosaccharide and its structure and microbial culture conditions and their media composition use are greatly dependent on the EPS-producing microorganisms [20]. In a study, Zhang et al. [143] reported a high molecular mass polysaccharide (1.1 × 10^6^ Da) [composed of glucose and galactose (2 : 1)] obtained from* Lb. plantarum* C88 (isolated from fermented dairy,* tofu*) when grown in a semidefined medium.* Lb. plantarum* (70810) screened from Chinese* Paocai* produced EPS with a narrow range of molecular mass (202.8–204.6 kDa) and is composed of three important monosaccharides (glucose, mannose, and galactose) [20]. Besides,* Lb. plantarum* KF5 isolated from Tibet* kefir* (traditional beverage recovered from fermentation of milk with kefir grains) was noted to be composed of similar types of monosaccharides as found in* Lb. plantarum* (70810) [144]. Gangoiti et al. [141] isolated an EPS producer strain (*Lb. plantarum* CIDCA 8327) from* kefir* with encouraging properties for the improvement of functional foods.* Lb. plantarum* K041 isolated from traditional Chinese pickle juice originating from Kaixian possessed high yield potential for EPS production [142]. There is voluminous research, which has revealed that some EPS produced by LAB have been considered as a potential grade of bioactive natural products in the biochemical and medical applications, such as immunomodulatory, antitumor, and antioxidant effects and cholesterol lowering activities [140, 145]. EPS from* Lb. plantarum* ZDY2013 may be a promising candidate for therapeutic and health food. The maximum yield was 429 ± 30.3 mg/L and the molecular mass was 5.17 × 10^4^ Da [33].

### 4.7. Biosurfactants

Microbial biosurfactants (BSs) are diverse group of amphiphilic compounds (both hydrophilic and hydrophobic moieties) produced by a variety of LAB species [146]. Bakhshi et al. [146] performed the screening of BSs formed by* Lb. plantarum* (PTCC 1896) based on dynamic surface tension (DST) values. One promising function of BSs is using them as an antiadhesive agent opposite to pathogenic bacteria [146].

### 4.8. Other Bioactive Compounds (Ascorbic Acid (AA), Total Phenols, etc.)

During the fermentation process, microorganisms can convert or degrade the phenolic compounds. Thus, by changing the structure of phenolic compounds, the complexation with iron and the bioavailability of the mineral can be influenced [4]. It is known that* Lb. plantarum*, a commonly used microorganism in plant based fermentation, contains tannase activity [147]. Gallic acid (GA) contains a galloyl group leading to complexation with iron and thus decreasing the availability of iron.* Lb. plantarum* LMG6907 is an efficient bacterium to destabilize the formed complexes which lead to an improved bioavailability of iron [147]. Composite organic waste/biowaste was assessed for its physical, chemical, and microbial suitability to serve as a substrate for the fermentative production of lactic acid (LA). In a study, Probst et al. [148] studied that the composite organic waste (biowaste), which is a preferred habitat of LAB* (Lb. plantarum)*, is used in a fermentation process for LA production.

## 5. Safety and Shelf-Life of Fermented Foods

**Figure 1:**
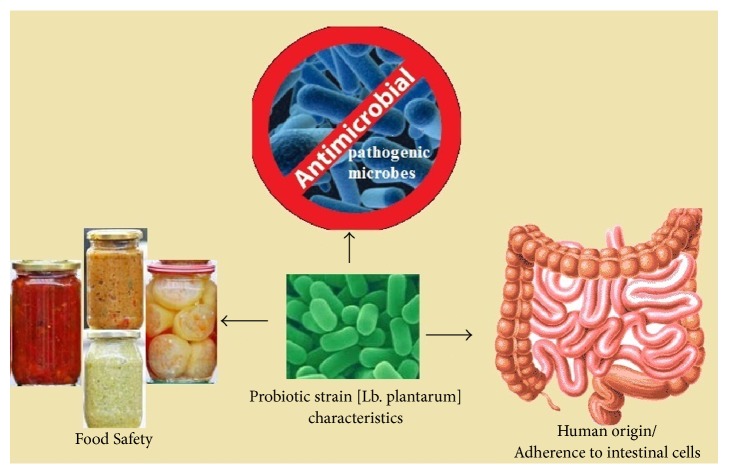
Theoretical basis for selection of* Lb. plantarum* strain. Use of probiotic bacteria* (Lb. plantarum)* is a useful strategy to obtain fermented food products with longer shelf-life (food safety) and safer properties due to their ability to delay or prevent the growth of common contamination bacteria (antimicrobial activity) and being of human origin as well as adhesion to intestinal cell lines.

Use of probiotic bacteria is a useful strategy to obtain products with longer shelf-life as well as safer properties due to their ability to delay or prevent the growth of common contamination bacteria [5, 115, 116] (Figure 1).

### 5.1. Bacteriocin

The bacteriocin is a wide range of genetically encoded antibacterial peptides, known to be active against closely related bacteria [149]. Some studies have also reported on activity against unrelated strains, especially those that are pathogenic and responsible for food spoilage [149].* Lb. plantarum* in particular produced bacteriocin of high activity and a wide range of antimicrobial activity including* S. aureus*,* L. monocytogenes*, and* A. hydrophila* [149, 150]. There are three approaches for potential application of* Lb. plantarum* strain and bacteriocin for biopreservation of foods in the food industry: inoculation of* Lb. plantarum* that produce the bacteriocin into foods during processing; application of the purified or crude bacteriocin directly onto the food product; and applications of a previously fermented product from a bacteriocin producing strain [9].

Barbosa et al. [23] isolated two-peptide plantaricin produced by* Lb. plantarum* (MBSa4) isolated from Brazilian salami. The molecular weight of bacteriocin produced by* Lb. plantarum* (MBSa4) was determined by SDS-PAGE to be around 2.5 kDa. A novel bacteriocin-M1-UVs300, which was produced by* Lb. plantarum* M1-UVs300, was purified and characterized from fermented sausage. Bacteriocin-M1-UVs300 was purified sequentially by an aqueous two-phase system (ATPS) and a Sephadex G-50 gel chromatography assay, combined with reverse phase high performance liquid chromatography (RE-HPLC) [125]. The molecular weight of the bacteriocin-M1-UVs300 was approximately 3.4 kDa, having major *β*-sheet (content of 52.43%), *α*-helix (16.17%), *β*-turn (15.27%), and random coil (16.12%). The bacteriocin-M1-UVs300 exhibited inhibitory activity against Gram-positive and Gram-negative bacteria. Also, it was relatively heat-resistant and it was also active over a range of pH (2–8), and it was sensitive to proteolytic enzymes, but not to *α*-amylase [125]. The bacteriocin withstands heating at 80°C for 120 min and is stored at 4°C for 6 months [150]. The bacteriocin formed by* Lb. plantarum* (MBSa4) confers stability to low pH and heat and is with long shelf-life. It is relevant to emphasize the antagonistic properties of* Lb. plantarum* (MBSa4) in contrast to fungi, which are natural spoilage organisms and can produce health-damaging mycotoxins [23]. The antilisterial bactericidal activity has also been proclaimed for other bacteriocin produced by* Lb. plantarum* [150]. Engelhardt et al. [151] examined the combining effect of common salt (NaCl) and low temperature on antilisterial bacteriocin production of* Lb. plantarum* (ST202Ch). The bacteriocin formation under high salt concentration and low temperature was found not adequate to restrict the growth of* Listeria monocytogenes*. Lin and Pan [9] characterized bacteriocin produced by* Lb. plantarum* (NTU 102) from homemade Korean style cabbage pickles. This strain exhibited good survival at an acidic condition (low pH), being vigor to high bile concentrations, increased tolerance/resistance to* Vibrio alginolyticus* infection, pathogen restriction, and good ability to cut back low-density lipoprotein (LDL-C) to high-density lipoprotein cholesterol (HDL-C) ratios.

### 5.2. Probiotic Properties of* Lb. plantarum* Strain

Over the last decades, the consumption of probiotics has attracted considerable attention. According to the FAO/WHO, probiotics spells “viable microorganisms that confer health benefits/aids to the host when administrated in adequate/competent amounts” [115, 116]. The scientific validity of* Lb. plantarum* strain as probiotics was first evaluated by characterizing bile and acid resistance (safeguard) in the intestinal tracts of animal and human hosts [35, 42]. Moreover,* Lb. plantarum* helped reduce overall symptoms of burden of infection of GI tracts [152]. It is believed that adherent probiotic* (Lb. plantarum)* has beneficial health effects, especially connected to the inhibition of pathogen adhesion to intestinal cell lines [22]. Two new strains, namely,* Lb. acidophilus* P110 and* Lb. plantarum* P164, were screened from faeces of healthy breast-fed (Egyptian) infants and were diagnosed as promising probiotics [153, 154]. In recent years, the curiosity in* Lb. plantarum* strain has heightened, chiefly in relation to its probiotic potential and its practicable application in variety of fermented foods and beverages [56, 107]. It is generally believed that the minimum concentration of living probiotic microorganism (*Lb. plantarum *strain) in the fermented food/product at the time of consumption should be at least 10^7^ CFU/ml (or/g) to achieve the proposed health benefits [155]. Jia et al. [14] isolated* Lb. plantarum* (KLDS1.0391) which is a probiotic strain from the traditional fermented dairy products and identified to produce bacteriocin opposing to Gram-negative and Gram-positive bacteria. Abushelaibi et al. [25] studied the investigation of probiotic characteristics and fermentation profile of selected LAB from raw camel milk.* Lb. plantarum* KX881772 and* Lb. plantarum* KX881779 appeared very promising in fermentation profiles. In addition,* Lb. plantarum* strain is recognized as natural probiotic of the human GI tract and can decrease intestinal heavy metal absorption, reduce metal accumulation in tissues, and alleviate hepatic oxidative stress [156]. Nevertheless, processing parameters/conditions, like pH, pressure, acidity, gastric acid, temperature, and bile salts, decrease the activity/viability of probiotic* (Lb. plantarum)* strain. Probiotics ought to be microencapsulated, in order that they are liberated in the GI tract in adequate numbers [43]. Various techniques for the microencapsulation of* Lb. plantarum* cells have been pursued as a form of cell protection [43, 157]. Among the materials used for microencapsulation of* Lb. plantarum*, the most frequently explored ones are chitosan, pectin, and natural gum (sodium alginate) [43, 157].

### 5.3. Antimicrobial Activity

*Lb. plantarum* strains are chief factors/components in a variety of fermentation processes whereby their fructification of organic acids, hydrogen peroxide (H_2_O_2_), diacetyl, and other antimicrobial components increased the safety and quality fermented foods [48]. LA is the major organic acid produced by* Lb. plantarum* strain. Other organic acids produced are acetic acid, propionic acid, phenyllactic acid (PLA), formic acid, and succinic acid. The approach of action of organic acids is the reduction of pH in the environment, causing inhibition of several microorganisms [66]. Guimarães et al. [139] demonstrated the potential use of* Lb. plantarum* (UM55) for inhibiting the growth of aflatoxigenic fungi* (Aspergillus flavus)*.* Lb. plantarum* (UM55) was analyzed for the existence of organic acids [e.g., lactic acid, phenyllactic acid (PLA), hydroxy phenyllactic acid (OH-PLA), and indole lactic acid (ILA)]. Lin and Pan [9] isolated* Lb. plantarum* (NTU 102) from homemade Korean style cabbage pickles. The antibacterial substances produced by* Lb. plantarum* (NTU 102), which is named LBP 102, exhibited a broad inhibitory spectrum. The remarkable effects of LBP 102 against this and other pathogens [*Vibrio parahaemolyticus* (BCRC 12864) and* Cronobacter sakazakii* (BCRC 13988)] indicated its potential as natural preservative. It is established that the electrostatic interactions with membrane of bacterial cells are authoritative/responsible for primary binding of antimicrobial agents [23]. Moreover, antimicrobial effect of organic acids is due to the undissociated form of organic acids. It can diffuse through the cell membrane, once internalized into the anions and protons. The proton ions impel the internal pH to decrease, resulting in interruption of proton motive force and preventing substrate transport mechanisms [66].

### 5.4. Antifungal Effects

Considering the harmful effects of fungi contamination several strategies to underrate mycotoxin production are of growing passion [158]. Antifungal activity of* Lb. plantarum *strains has been exhibited to be due to presence of phenyllactic acid (PLA), cyclic dipeptides, fatty acids, and organic acids [48]. Gupta and Srivastava [159] studied the antifungal effect of antimicrobial peptides (AMPs LR14) caused by* Lb. plantarum* (LR14) in contrast to spoilage fungi (e.g.,* Aspergillus niger, Mucor racemosus, Penicillium chrysogenum*, and* Rhizopus stolonifer*). The peptides (AMPs LR14) caused dysfunction to both the hyphal growth and spore germination of fungi. However, in food industry, fermented food systems containing fungal spoilage (microorganisms) can be diminished by the supplementation of appropriate amount salts and propanoic acid. In addition, adoption of modified atmosphere packaging and biopreservation principles (e.g., pasteurization or irradiation) are essential for increasing the effects of antifungal effects [23]. More recently, Dong et al. [48] suggested that protein and/or carbohydrate moiety of LAB (*Lb. plantarum* strain) show a major act in mycotoxin binding/attaching. Nevertheless, the mechanism of antifungal response is difficult to unfold/illustrate due to complicated and cooperative interactions between these considerable groups of antimicrobial compounds (e.g., peptides, proteins, and organic acids) [23].

### 5.5. Antioxidant Properties

*Lb. plantarum* strains screened from conventional fermented food possess many functional properties, especially antioxidant properties [160]. They are colonized in the intestinal tract and play a critical role in protection from free radicals. In addition, antioxidant activity of* Lb. plantarum* strains contributes to the preservation of various disorders (e.g., diabetes, cardiovascular diseases, and ulcers of GI tract) [160]. Yadav [37] studied the antioxidant properties of chicken sausages (prepared from minced chicken meat) fermentation with* Lb. plantarum* supplemented with starch and dextrose as well as evaluation of scavenging activity across 2-2-azino-bis-3 ethylbenzthiazoline-6-sulphonic acid (ABTS) radical cation, superoxide anion (SASA), 1,1-diphenyl-2-picrylhydrazyl (DPPH) free radicals, lipid oxidation, and thiobarbituric acid (TBA value). Tang et al. [161] isolated* Lb. plantarum* (MA2) strain and evaluated the antioxidant activities (*in vitro* strain) from Chinese traditional Tibetan* kefir* grains. The results revealed that* Lb. plantarum* (MA2) can accept hydrogen peroxide (H_2_O_2_, >2.0 mM) and its fermentate had strong/potent reducing ability and free radical scavenging capacity. Moreover, three groups of antioxidant-related genes (*cat*,* gshR*, and* npx*) were found upregulated under H_2_O_2_ challenge.

### 5.6. Antimutagenic Activity

It has been shown that specific probiotic strains exert antiproliferative effects via collective actions between adhesion/attachment to colon malignant cells and production of fatty acids, mainly butyric and propionic acids [162]. Saxami et al. [32] studied the modes of action and the potential beneficial effects of* Lb. plantarum* B282 on human colorectal cancer cells. The strain exhibited significantly higher adhesion rates and inhibited growth of human colon cancer cells (Caco-2 colon and HT-29) by promoting a G1 phase arrest and downregulation of specific* cyclin* genes.

### 5.7. Immune Response

*Lactobacillus *sp. being the most commonly used probiotic agent improves intestinal microbiota and gut health and regulates immune system in consumers [163]. Several reports suggested that the supplementation of probiotics (*Lb. plantarum *strain) can improve the growth, disease resistance, and immune response of fish [164, 165]. Considering the tolerance-inducing immunomodulatory effects of* Lb. plantarum*, it is of attraction/interest to search the opportunity of using allergen expressing* Lactobacillus* as delivery vehicle in immunotherapy or an allergy vaccine [166]. Minic et al. [166] studied analysis of the prospect to use engineered* Lb. plantarum* (WCFS1) with a surface-displayed respiratory allergen (Fes p1) in immunotherapy for pollen allergy.* Lb. plantarum* showed an increased level of specific serum IgA. The recent studies demonstrated that* Lb. plantarum* CCFM639, a selected candidate probiotic strain with enhanced aluminium- (Al-) binding, antioxidative, and immunomodulatory abilities* in vitro* and* in vivo*, provides significant protection against Al-toxicity in mice [167]. In a study, Kwon et al. [168] reported that the anti-inflammatory effect of* Bifidobacterium longum* LC67 and* Lb. plantarum* LC27 isolated from* kimchi*. These strains induced interleukin- (IL-) 8 and tumor necrosis factor (TNF) expression and stimulated macrophages against ethanol-induced gastritis and liver injury in mice [168].

### 5.8. Health-Promoting Properties

The increasing health awareness with the use/consumption of probiotic strains has been encouraged among consumers, to overcome the growing diseases risks [e.g., intolerance to lactose, diabetes, and cardiovascular diseases (CVDs)] in the world nowadays [22]. Recently,* Lb. plantarum* has been applied in medical fields for the cure of different chronic and CVDs (e.g., Alzheimer's, Parkinson's, diabetes, obesity, cancer, hypertension, urinogenital complications, and liver disorders) [169].* In vitro* studies examining various cell lines have indicated that* Lb. plantarum* strains have a therapeutic effect [170]. Furthermore, clinical analyses have shown the efficacy of* Lb. plantarum* strains in the cure or treatment of gastrointestinal disorders, along with irritable bowel syndrome and ulcerative colitis, including diarrheal diseases (e.g., antibiotic-associated diarrhea and* Clostridium difficile*-associated diarrhea) [170, 171]. For instance,* kimchi* is familiar as a healthy food and provides the health-promoting effects (e.g., anticancer, antioxidative, antidiabetic, and antiobesity effects) [135]. Park et al. [3] isolated* Lb. plantarum* (HAC01) from white* kimchi* and gave it to a diet-induced obese (DIO) mouse that received a high-fat (HF) diet to assess the functionality* in vivo*. The mouse received the life/viable strains which revealed great decrease in body weight and total weight gain during 8 weeks compared to the high-fat control group. More recently, Mihailović et al. [172] studied assessing the effect of the probiotic* Lb. paraplantarum* BBCG11 on the regulatory pathway underlying the defense responses of the liver and kidney in diabetic rats. The probiotic (*Lb. paraplantarum* BBCG11) administration found the development of diabetic complications in rats. El Temsahy et al. [154] studied the defensive efficiency of new safe probiotic strains [*Lb. plantarum* (P164) and* Lb. acidophilus* (P110)] screened from faeces of breast-fed infants, against experimental trichinelllosis in mice.* Lb. plantarum* P164 induced a noticeable parasitological and histopathological improvement toward* Trichinella* infestation in mice. Thus, this promising probiotic strain contributes a future preventive scope as a possible safe natural protective agent against* T. spiralis* infection.

## 6. Future Research Focus

The scope that needs vigilant study is the probiotic (*Lb. plantarum* strain) inclusion along with the use of protein supplement in starch-/nondairy-based fermented food/beverages for challenging prevalent protein-energy malnutrition in the world. The incorporation of probiotics* (Lb. plantarum)* strains into traditional fermented food system is presently being investigated as a way of recommending the rural community to resolve worsening health conditions. In addition, education/training and awareness/enlightenment of provincial/local processors and consumers with upgrading and optimization of local technologies would go a long way to sustain the health and functional benefits of such starch-/nondairy-based fermented food/beverages in developing countries.

## 7. Conclusion

*Lb. plantarum* strains were obtained from indigenous fermented foods and involved in the fermentation of nondairy and dairy products/foods. These strains retain a momentous capability to contrast various pathogenic bacteria, including both Gram-negative and Gram-positive species, which can contaminate food and are responsible for diseases in humans. The biosynthesis of organic acids, enzyme systems, bioactive peptides, vitamins, and EPS is proposed as one of the main mechanisms through which the antimicrobial, antioxidant, and probiotic activities are exerted. The antagonistic feature and probiotic properties of* Lb. plantarum* strains can be a distinctive trait/function as biocontrol agents against potentially harmful microorganisms during food processing and storage and also increased the shelf-life and safety of fermented foods. To reduce the use of chemical compounds, this probiotic strain present in fermented food system can contribute/warrant increasing health and well-being and reducing the risk of the consumer. There are still many challenges ahead; and in any case the choice of probiotic strain to be used in fermented food is essential.

## Figures and Tables

**Table 1:** Molecular methods/techniques used for identification of *Lb. plantarum* strains.

Molecular method	Primer used	Primer sequence (5′-3′)	Identified * Lb. plantarum* strain	Reference
PCR	LbPI1 (forward) and LbPI2 (reverse)	CCG TTT ATG CGG AAC ACC TA and TCG GGA TTA CCA AAC ATC AC	*Lb. plantarum* ATCC 8014	Quere et al. [18]
RAPD-PCR	16S rRNA-based primer, P32	TAC CAC TAC AAT GGA TG	*Lb. plantarum* ATCC 14917	Elegado et al. [19]
RAPD-PCR	16S rRNA-based primer, A27F	AGC GGA TCA CTT CAC ACA GGA CTA CGG CTA CCT TGT TAC GA	*Lb. plantarum* YW11	Wang et al. [20]
RAPD-PCR	UB16S-F and UB16S-R	AGA GTT TGA TCC TGG CTC AG and ACG GCT ACC TTG TTA CGA CT	*Lb. plantarum* NTMI05 and NTMI20	Imran et al. [21]
16S rDNA sequencing	Universal primer, SSU	TGC CAG CAG CCG CGG TA and GAC GGG CGG TGT GTA CAA	*Lb. plantarum* B128, B134, B143, B149, B166, B174	Mahmoudi et al. [22]
16S rDNA sequencing	8f and 1512r	CAC GGA TCC AGA CTT TGA T(C/T)(A/C) TGG CTC AG and GTG AAG CTT ACG G(C/T)T AGC TTG TTA CGA CTT	*Lb. plantarum* MBSa4	Barbosa et al. [23]
RAPD and PFGE^*∗*^	OPA5 and OPA20	AAT CGG GCT G and GTT GCG TCC	*Lb. plantarum* ATCC 8014 and * Lb. plantarum* SD1S612	Adesulu-Dahunsi et al. [24]
16S rDNA sequencing	27F and 1492R	AGA GTT TGA TCC TGG CTC AG and TAC GGY TAC CTT GTT ACG ACT T	*Lb. plantarum* KX881772 and *Lb. plantarum* KX881779	Abushelaibi et al. [25]
ITS-PCR	16SF-R2 and 23SR-R10	AGA GTT TGA TCC TGG CTC AG and AAG GAG GTG ATC CAG CCG CA	*Lb. plantarum* GA106, FU137, NRRLB-14768, DSM10667, JCM1558, DK0–22, OB123, OF101, YO175	Adesulu-Dahunsi et al. [24]

RAPD-PCR: random amplified polymorphic DNA-polymerase chain reaction; PCR: polymerase chain reaction; PFGE: pulsed-field gel electrophoresis; ITS-PCR: 16S–23S rRNA gene intergenic transcribed spacer PCR amplification. ^**∗**^Restriction enzyme (*ApaI* and *SfiI*).

**Table 2:** *Lactobacillus plantarum* strain mediated fermented food products.

Fermented foods	Fermentable substrate/source	Identified * Lb. plantarum* strain	Special features/application	Reference
Traditional fermented foods				
*Chourico* ^+^	-	*Lb. plantarum* DSMZ 12028	Inducing proinflammatory response	Cammarota et al. [29]
*Tofu*	Chinese fermented dairy	*Lb. plantarum* C88	Antioxidant activity	Li et al. [30]
*Fufu*	Cassava (*Manihot esculenta* Crantz) flour	*Lb. plantarum* strain 6710	Protein-fortified product	Rosales-Soto et al. [31]
*White (Baek) kimchi*	Chinese cabbage without chili	*Lb. plantarum* HAC01	New probiotic development	Park et al. [3]
*Fermented table olives*	Spanish-style green olives	*Lb. plantarum *B282	Adhesion and antiproliferative effects of colorectal cancer cells	Saxami et al. [32]
*Acid beans*	*Vigna unguiculata*	*Lb. plantarum* ZDY2013	EPS	Zhang et al. [33]
*Kimchi *	Chinese cabbage	Enriched with *Lb. plantarum* Ln4	Probiotic effect	Son et al. [34]
*Kimchi*	Baechu (napa cabbage)	*Lb. plantarum* wikim 18 (KFCC 1188P)	Probiotic effect	Jung et al. [35]
*Korean kimchi *		*Lb. plantarum* LBP-K10	Antimicrobial activity	Kwak et al. [36]
*Cabbage pickle *	Korean cabbage	Cell-free supernatant of *Lb. plantarum* NTU 102	Effective against *V. parahaemolyticus* BCRC 12864 and *Cronobacter sakazakii* BCRC 13988	Lin and Pan [9]
*Chicken sausage*	Minced meat	*Lb. plantarum*	Antioxidant activity	Yadav, [37]
*Kunu* ^*ψ*^	Millet *(Pennisetum glaucum)*	*Lb. reuteri, Lb. plantarum, *and* Lb. acidophilus*	Enhanced nutrient qualities, shelf-life, and antioxidant potentials	Adedire et al. [38]
*Pickled cabbage *	Cabbage	*Lb. plantarum* ATCCI 4917	-	Turpin et al. [39]

Novel fermented foods				
*Fermented oat food*	-	*Lb. plantarum *UFG9; *Lb. plantarum *B2	Increased riboflavin (VitB2) concentration	Russo et al. [40]
*Fermented soymilk *	Soybean	*Lb. plantarum *TWK10	Antimelanogenic property	
*Pinot noir wine (Patagonian red wines)*	-	*Lb. plantarum *ATCC 14917	Malolactic starter cultures	Brizuela et al. [41]
*FRGE*	Korean ginseng (*Panax ginseng* Meyer)	*Lb. plantarum *KCCM 11613P	Antioxidant activity	Jung et al. [42]
*Litchi juice*	Litchi (*Litchi chinensis* Sonn.)	Spray drying of probiotic bacteria (*Lb. plantarum* MTCC 2621) with prebiotics^*∗*^	Stimulated the digestive system	Kalita et al. [43]
*Bread*	-	*Lb. plantarum* P8	Improved baking conditions and storage	Zhang et al. [44]

FRGE: fermented red ginseng extract; EPS: exopolysaccharide.^**∗**^Fructooligosaccharide (FOS), inulin, gum arabic, and pectin. ^+^Portuguese dry fermented sausage. ^*ψ*^Nonalcoholic beverage of Nigeria.

## Abbreviations

ABTS: 2-2-Azino-bis-3 ethylbenzthiazoline-6-sulphonic acid
CVDs: Cardiovascular diseases
EMP: Embden-Meyerhof-Parnas pathway
EPS: Exopolysaccharide
FAO: Food and Agriculture Organization
GC: Gas chromatography
GC-MS: Gas chromatography and mass spectrometry
GI: Gastrointestinal tract
GRAS: Generally recognized as safe
HPLC: High performance liquid chromatography
IL: Interleukin
IS: Insertion sequence
LAB: Lactic acid bacteria
LPG: * Lb. plantarum*-group
MLVA: Multilocus variable number tandem repeat analysis
NMR: Nuclear magnetic resonance
ORFs: Open reading frames
PCR: Polymerase chain reaction
PFGE: Pulsed-field gel electrophoresis
PK: Phosphoketolase pathway
QPS: Qualified presumption of safety
TBA: Thiobarbituric acid
TNF: Tumor necrosis factor
VBNC: Viable but nonculturable
WHO: World Health Organization.

## References

[1] Axelsson L., Ahrné S., Ahrné S. (2000). Lactic acid bacteria. *Applied Microbial Systematics*.

[2] Ray R. C., Joshi V. K., Ray R. C., Montet D. (2014). Fermented Foods: Past, present and future scenario. *Microorganisms and Fermentation of Traditional Foods*.

[3] Park S., Ji Y., Park H. (2016). Evaluation of functional properties of lactobacilli isolated from Korean white kimchi. *Food Control*.

[4] Ray R. C., Panda S. H., Palino M. V. (2007). Lactic acid fermented fruits and vegetables: an overview. *Food Microbiology Research Trends*.

[5] Panda S. H., Kar N. B., Ray R. C., Montet D., Sayyed R. Z., Patil A. S. (2008). Probiotic lactic acid bacteria: applications in food, feed and pharmaceutical industries. *Biotechnology Emerging Trends*.

[6] Behera S. S., Ray R. C., Rosell C. M., Bajerska J., El Sheikha A. F. (2015). Sourdough bread. *Bread Fortification for Nutrition and Health*.

[7] Drosinos E. H., Paramithiotis S. (2017). Current trends in microbiological, technological and nutrional aspects of fermented sausages. *Fermented Foods. Part II. Technological Interventions*.

[8] Saigal D., Ray R. C., Ray R. C., Ward O. P. (2007). Winemaking: Microbiology, biochemistry and biotechnology. *Microbial Biotechnology in Horticulture*.

[9] Lin T.-H., Pan T.-M. (2017). Characterization of an antimicrobial substance produced by *Lactobacillus plantarum* NTU 102. *Journal of Microbiology, Immunology and Infection*.

[10] Elagöz A., Abdi A., Hubert J.-C., Kammerer B. (1996). Structure and organisation of the pyrimidine biosynthesis pathway genes in *Lactobacillus plantarum*: A PCR strategy for sequencing without cloning. *Gene*.

[11] Liu B., Yang M., Qi B., Chen X., Su Z., Wan Y. (2010). Optimizing l-(+)-lactic acid production by thermophile *Lactobacillus plantarum* As. 1.3 using alternative nitrogen sources with response surface method. *Biochemical Engineering Journal*.

[12] Guidone A., Zotta T., Ross R. P. (2014). Functional properties of *Lactobacillus plantarum* strains: A multivariate screening study. *LWT-Food Science and Technology*.

[13] Ricci A., Allende A., Bolton D. (2017). Update of the list of QPS‐recommended biological agents intentionally added to food or feed as notified to EFSA 5: suitability of taxonomic units notified to EFSA until September 2016. *EFSA Journal*.

[14] Jia F.-F., Zhang L.-J., Pang X.-H. (2017). Complete genome sequence of bacteriocin-producing *Lactobacillus plantarum* KLDS1. 0391, a probiotic strain with gastrointestinal tract resistance and adhesion to the intestinal epithelial cells. *Genomics*.

[15] Ray R. C., Sivakumar P. S. (2009). Traditional and novel fermented foods and beverages from tropical root and tuber crops: review. *International Journal of Food Science & Technology*.

[16] Freire A. L., Ramos C. L., da Costa Souza P. N., Cardoso M. G. B., Schwan R. F. (2017). Nondairy beverage produced by controlled fermentation with potential probiotic starter cultures of lactic acid bacteria and yeast. *International Journal of Food Microbiology*.

[17] Dellaglio F., Felis G. E. (2005). Taxonomy of lactobacilli and bifidobacteria. *Probiotics and Prebiotics: Scientific Aspects*.

[18] Quere F., Deschamps A., Urdaci M. C. (1997). DNA probe and PCR-specific reaction for *Lactobacillus plantarum*. *Journal of Applied Microbiology*.

[19] Elegado F. B., Guerra M. A. R. V., Macayan R. A., Mendoza H. A., Lirazan M. B. (2004). Spectrum of bacteriocin activity of *Lactobacillus plantarum* BS and fingerprinting by RAPD-PCR. *International Journal of Food Microbiology*.

[20] Wang J., Zhao X., Tian Z., Yang Y., Yang Z. (2015). Characterization of an exopolysaccharide produced by *Lactobacillus plantarum* YW11 isolated from Tibet Kefir. *Carbohydrate Polymers*.

[21] Imran M. Y. M., Reehana N., Jayaraj K. A. (2016). Statistical optimization of exopolysaccharide production by *Lactobacillus plantarum* NTMI05 and NTMI20. *International Journal of Biological Macromolecules*.

[22] Mahmoudi I., Moussa O. B., Khaldi T. E. M. (2016). Functional in vitro screening of *Lactobacillus* strains isolated from Tunisian camel raw milk toward their selection as probiotic. *Small Ruminant Research*.

[23] Barbosa M. S., Todorov S. D., Ivanova I. V. (2016). Characterization of a two-peptide plantaricin produced by *Lactobacillus plantarum* MBSa4 isolated from Brazilian salami. *Food Control*.

[24] Adesulu-Dahunsi A. T., Sanni A. I., Jeyaram K., Banwo K. (2017). Genetic diversity of *Lactobacillus plantarum* strains from some indigenous fermented foods in Nigeria. *LWT- Food Science and Technology*.

[25] Abushelaibi A., Al-Mahadin S., El-Tarabily K., Shah N. P., Ayyash M. (2017). Characterization of potential probiotic lactic acid bacteria isolated from camel milk. *LWT- Food Science and Technology*.

[26] Gu C. T., Wang F., Li C. Y., Liu F., Huo G. C. (2012). *Lactobacillus xiangfangensis* sp. nov., isolated from Chinese pickle. *International Journal of Systematic and Evolutionary Microbiology*.

[27] Huang C.-H., Chang M.-T., Huang L. (2014). Cloning of a novel specific SCAR marker for species identification in *Lactobacillus pentosus*. *Molecular and Cellular Probes*.

[28] Devi S. M., Aishwarya S., Halami P. M. (2016). Discrimination and divergence among *Lactobacillus plantarum*-group (LPG) isolates with reference to their probiotic functionalities from vegetable origin. *Systematic and Applied Microbiology*.

[29] Cammarota M., de Rosa M., Stellavato A., Lamberti M., Marzaioli I., Giuliano M. (2009). In vitro evaluation of *Lactobacillus plantarum* DSMZ 12028 as a probiotic: emphasis on innate immunity. *International Journal of Food Microbiology*.

[30] Li S., Zhao Y., Zhang L. (2012). Antioxidant activity of *Lactobacillus plantarum* strains isolated from traditional Chinese fermented foods. *Food Chemistry*.

[31] Rosales-Soto M. U., Gray P. M., Fellman J. K. (2016). Microbiological and physico-chemical analysis of fermented protein-fortified cassava (*Manihot esculenta* Crantz) flour. *LWT- Food Science and Technology*.

[32] Saxami G., Karapetsas A., Lamprianidou E. (2016). Two potential probiotic lactobacillus strains isolated from olive microbiota exhibit adhesion and anti-proliferative effects in cancer cell lines. *Journal of Functional Foods*.

[33] Zhang Z., Liu Z., Tao X., Wei H. (2016). Characterization and sulfated modification of an exopolysaccharide from *Lactobacillus plantarum* ZDY2013 and its biological activities. *Carbohydrate Polymers*.

[34] Son S.-H., Jeon H.-L., Jeon E. B. (2017). Potential probiotic *Lactobacillus plantarum* Ln4 from kimchi: evaluation of *β*-galactosidase and antioxidant activities. *LWT-Food Science and Technology*.

[35] Jung J., Jang H. J., Eom S. J., Choi N. S., Lee N.-K., Paik H.-D. (2017). Fermentation of red ginseng extract by the probiotic *Lactobacillus plantarum* KCCM 11613P: Ginsenoside conversion and antioxidant effects. *Journal of Ginseng Research*.

[36] Kwak M. K., Liu R., Kang S. O. (2017). Antimicrobial activity of cyclic dipeptides produced by *Lactobacillus plantarum* LBP-K10 against multidrug-resistant bacteria, pathogenic fungi, and influenza A virus. *Food Control*.

[37] Yadav A. S. (2017). Antioxidant and antimicrobial profile of chicken sausages prepared after fermentation of minced chicken meat with Lactobacillus plantarum and with additional dextrose and starch. *LWT- Food Science and Technology*.

[38] Adedire O. M., Farinu A. O., Olaoye S. O., Osesusi A. O., Ibrahim K. O. (2017). The Effect of Enhanced Fermentation on the Antioxidant, Proximate and Shelf Life Properties of Kunu. *American Journal of Biology and Life Sciences*.

[39] Turpin W., Weiman M., Guyot J.-P., Lajus A., Cruveiller S., Humblot C. (2017). The genomic and transcriptomic basis of the potential of *Lactobacillus plantarum* A6 to improve the nutritional quality of a cereal based fermented food. *International Journal of Food Microbiology*.

[40] Russo P., de Chiara M. L. V., Capozzi V. (2016). *Lactobacillus plantarum* strains for multifunctional oat-based foods. *LWT- Food Science and Technology*.

[41] Brizuela N. S., Bravo-Ferrada B. M., La Hens D. V. (2017). Comparative vinification assays with selected Patagonian strains of *Oenococcus oeni* and *Lactobacillus plantarum*. *LWT-Food Science and Technology*.

[42] Jung M. Y., Lee J., Park B. (2017). Applicability of a colorimetric method for evaluation of lactic acid bacteria with probiotic properties. *Food Microbiology*.

[43] Kalita D., Saikia S., Gautam G., Mukhopadhyay R., Mahanta C. L. (2018). Characteristics of synbiotic spray dried powder of litchi juice with *Lactobacillus plantarum* and different carrier materials. *LWT- Food Science and Technology*.

[44] Zhang L., Taal M. A., Boom R. M., Chen X. D., Schutyser M. A. I. (2018). Effect of baking conditions and storage on the viability of *Lactobacillus plantarum* supplemented to bread. *LWT- Food Science and Technology*.

[45] Heinl S., Grabherr R. (2017). Systems biology of robustness and flexibility: *Lactobacillus buchneri*—A show case. *Journal of Biotechnology*.

[46] Pérez-Díaz I. M., Hayes J., Medina E. (2017). Reassessment of the succession of lactic acid bacteria in commercial cucumber fermentations and physiological and genomic features associated with their dominance. *Food Microbiology*.

[47] Williams J. G., Hanafey M. K., Rafalski J. A., Tingey S. V. (1995). Genetic analysis using random amplified polymorphic DNA markers. *Recombinant DNA Methodology II*.

[48] Dong A.-R., Lo R., Bansal N., Turner M. S. (2017). A genetic diversity study of antifungal *Lactobacillus plantarum* isolates. *Food Control*.

[49] Liu C.-J., Wang R., Gong F.-M. (2015). Complete genome sequences and comparative genome analysis of *Lactobacillus plantarum* strain 5-2 isolated from fermented soybean. *Genomics*.

[50] Zhang W., Sun Z., Bilige M., Zhang H. (2015). Complete genome sequence of probiotic *Lactobacillus plantarum* P-8 with antibacterial activity. *Journal of Biotechnology*.

[51] Kim Y., Ryu B. H., Kim J. (2017). Characterization of a novel SGNH-type esterase from *Lactobacillus plantarum*. *International Journal of Biological Macromolecules*.

[52] Delgado S., Flórez A. B., Guadamuro L., Mayo B. (2017). Genetic and biochemical characterization of an oligo-*α*-1,6-glucosidase from *Lactobacillus plantarum*. *International Journal of Food Microbiology*.

[53] Emser K., Barbosa J., Teixeira P., Bernardo de Morais A. M. M. (2017). *Lactobacillus plantarum* survival during the osmotic dehydration and storage of probiotic cut apple. *Journal of Functional Foods*.

[54] Food and Agriculture Organization (2002). *Guidelines for the evaluation of probiotics in food: Report of a Joint FAO/WHO Working Group on Drafting Guidelines for the Evaluation of Probiotics in Food*.

[55] Swain M. R., Anandharaj M., Ray R. C., Parveen Rani R. (2014). Fermented fruits and vegetables of Asia: a potential source of probiotics. *Biotechnology Research International*.

[56] Montet D., Ray R. C., Zakhia-Rozis N., Ray R. C., Montet D. (2014). Lactic acid fermentation of vegetables and fruits. *Microorganisms and Fermentation of Traditional Foods*.

[57] Panda S. H., Ray R. C. (2007). Lactic acid fermentation of *β*-carotene rich sweet potato (*Ipomoea batatas* L.) into lacto-juice. *Plant Foods for Human Nutrition*.

[58] Panda S. H., Parmanick M., Ray R. C. (2007). Lactic acid fermentation of sweet potato (*Ipomoea batatas* L.) into pickles. *Journal of Food Processing and Preservation*.

[59] Panda S. H., Naskar S. K., Sivakumar P. S., Ray R. C. (2009). Lactic acid fermentation of anthocyanin‐rich sweet potato (*Ipomoea batatas* L.) into lacto‐juice. *International Journal of Food Science & Technology*.

[60] Panda S. H., Panda S., Sethuraman Sivakumar P., Ray R. C. (2009). Anthocyanin- rich sweet potato lacto- pickle: Production, nutritional and proximate composition. *International Journal of Food Science & Technology*.

[61] Panda S. H., Naskar S. K., Ray R. C. (2006). Production, proximate and nutritional evaluation of sweet potato curd. *Journal of Food, Agriculture and Environment (JFAE)*.

[62] Mohapatra S., Panda S. H., Sahoo S. K., Sivakumar P. S., Ray R. C. (2007). *β*-Carotenerich sweet potato curd: production, nutritional and proximate composition. *International Journal of Food Science & Technology*.

[63] Ji Y., Kim H., Park H. (2013). Functionality and safety of lactic bacterial strains from Korean kimchi. *Food Control*.

[64] Blana V. A., Polymeneas N., Tassou C. C., Panagou E. Z. (2016). Survival of potential probiotic lactic acid bacteria on fermented green table olives during packaging in polyethylene pouches at 4 and 20°C. *Food Microbiology*.

[65] Abadi Sherahi M. H., Shahidi F., Yazdi F. T., Hashemi S. M. B. (2017). Effect of *Lactobacillus plantarum* on olive and olive oil quality during fermentation process. *LWT-Food Science and Technology*.

[66] Saelim K., Jampaphaeng K., Maneerat S. (2017). Functional properties of *Lactobacillus plantarum* S0/7 isolated fermented stinky bean (Sa Taw Dong) and its use as a starter culture. *Journal of Functional Foods*.

[67] Bomrungnok W., Sonomoto K., Pinitglang S., Wongwicharn A. (2012). Single step lactic acid production from cassava starch by *Lactobacillus plantarum* sw14 in conventional continuous and continuous with high cell density. *APCBEE Procedia*.

[68] Mukisa I. M., Byaruhanga Y. B., Muyanja C. M. B. K., Langsrud T., Narvhus J. A. (2017). Production of organic flavor compounds by dominant lactic acid bacteria and yeasts from Obushera, a traditional sorghum malt fermented beverage. *Food Science & Nutrition*.

[69] Nyanzi R., Jooste P. J., Makun H. A. (2012). Cereal based functional foods. *E. C. Rigobelo Processes and Prospects. pp. 153–179*.

[70] Settanni L., Tanguler H., Moschetti G., Reale S., Gargano V., Erten H. (2011). Evolution of fermenting microbiota in tarhana produced under controlled technological conditions. *Food Microbiology*.

[71] Evans E., Musa A., Abubakar Y., Mainuna B. (2013). *Nigerian Indigenous Fermented Foods: (ed.). Probiotics*.

[72] Muyanja C. M. B. K., Narvhus J. A., Treimo J., Langsrud T. (2003). Isolation, characterisation and identification of lactic acid bacteria from bushera: A Ugandan traditional fermented beverage. *International Journal of Food Microbiology*.

[73] Zorba M., Hancioglu O., Genc M., Karapinar M., Ova G. (2003). The use of starter cultures in the fermentation of boza a traditional Turkish beverage. *Process Biochemistry*.

[74] Cosansu S. (2009). Determination of biogenic amines in a fermented beverage, boza. *Journal of Food, Agriculture and Environment (JFAE)*.

[75] Kivanc M., Yilmaz M., Caktr E. (2011). Isolation and identifi cation of lactic acid bacteria from boza, and their microbial activity against several reporter strains. *Turkish Journal of Biology*.

[76] Das A., Raychaudhuri U., Chakraborty R. (2012). Cereal based functional food of Indian subcontinent: a review. *Journal of Food Science and Technology*.

[77] Karovičová Z. K. J. (2007). Fermentation of cereals for specific purpose. *Journal of Food and Nutrition Research*.

[78] Champagne C. P., Charalampopoulos D., Rastall R. A. (2009). Some technological challenge sin the addition of probiotic bacteria to foods. *Prebiotics and Probiotics Science and Technology*.

[79] Fraqueza M. J., Patarata L., Laukova A., Zdolec N. (2017). Protective cultures and bacteriocins in fermented meats. *Fermented Meat Products: Health Aspects*.

[80] Albano H., van Reenen C. A., Todorov S. D. (2009). Phenotypic and genetic heterogeneity of lactic acid bacteria isolated from ‘Alheira’, a traditional fermented sausage produced in Portugal. *Meat Science*.

[81] Drosinos E. H., Mataragas M., Xiraphi N., Moschonas G., Gaitis F., Metaxopoulos J. (2005). Characterization of the microbial flora from a traditional Greek fermented sausage. *Meat Science*.

[82] Kozačinski L., Zdolec N., Hadžiosmanović M., Cvrtila Ž., Filipović I., Majić J. (2006). Microbial flora of the Croatian traditional fermented sausage. *Archiv fur Lebensmittelhygiene*.

[83] Aquilanti L., Santarelli S., Silvestri G., Osimani A., Petruzzelli A., Clementi F. (2007). The microbial ecology of a typical Italian salami during its natural fermentation. *International Journal of Food Microbiology*.

[84] Zdolec N., Dobranić V., Horvatić A., Vučinić S. (2013). Selection and application of autochthonous functional starter cultures in traditional Croatian fermented sausages. *International Food Research Journal*.

[85] Danilović B., Savić D., Zdolec N. (2017). Microbial ecology of fermented sausages and dry-cured meats. *Fermented Meat Products: Health Aspects*.

[86] Rai A. K., Tamang J. P., Palni U. (2010). Microbiological studies of ethnic meat products of the Eastern Himalayas. *Meat Science*.

[87] Wanangkarn A., Liu D.-C., Swetwiwathana A. (2014). Lactic acid bacterial population dynamics during fermentation and storage of Thai fermented sausages according to restriction fragment lenght polymorphism analysis. *International Journal of Food Microbiology*.

[88] Antara N. S., Sujaya I. N., Yokota A., Asano K., Aryanta W. R., Tomita F. (2002). Identification and succession of lactic acid bacteria during fermentation of ‘urutan’, a Balinese indigenous fermented sausage. *World Journal of Microbiology and Biotechnology*.

[89] Mejri L., Hassouna M. (2016). Characterization and selection of *Lactobacillus plantarum* species isolated from dry fermented sausage reformulated with camel meat and hump fat. *Applied Biological Chemistry*.

[90] Frece J., Kovačević D., Kazazić S. (2014). Comparison of sensory properties, shelf-life and microbiological safety of industrial sausages produced with autochthonous and commercial starter cultures (Starter cultures for sausages production). *Food Technology and Biotechnology*.

[91] da Silva Sabo S., Vitolo M., Dominguez Gonzales J. M., de Souza Oliveira R. P. (2014). Overview of *Lactobacillus plantarum* as a promising bacteriocin producer among lactic acid bacteria. *Food Research International*.

[92] Holck A., Axelsson L., McLeod A., Rode T. M., Heir E. (2017). Health and safety considerations of fermented sausages. *Journal of Food Quality*.

[93] Laranjo M., Elias M., Fraqueza M. J. (2017). The Use of Starter Cultures in Traditional Meat products. *Journal of Food Quality*.

[94] Zdolec N. (2017). *Fermented Meat Products: Health Aspects*.

[95] Työppönen S., Markkula A., Petäjä E., Suihko M.-L., Mattila-Sandholm T. (2002). Survival of *Listeria monocytogenes* in North European type dry sausages fermented by bioprotective meat starter cultures. *Food Control*.

[96] Sun Q., Chen Q., Li F., Zheng D., Kong B. (2016). Biogenic amine inhibition and quality protection of Harbin dry sausages by inoculation with *Staphylococcus xylosus* and *Lactobacillus plantarum*. *Food Control*.

[97] Rubio R., Jofré A., Aymerich T., Guàrdia M. D., Garriga M. (2014). Nutritionally enhanced fermented sausages as a vehicle for potential probiotic lactobacilli delivery. *Meat Science*.

[98] Blaiotta G., Murru N., Di Cerbo A., Romano R., Aponte M. (2018). Production of probiotic bovine salami using *Lactobacillus plantarum* 299v as adjunct. *Journal of the Science of Food and Agriculture*.

[99] Nova R. J., Botsaris G., Cerda-Leal F., Zdolec N. (2017). Probiotics in fermented meat products. *Fermented Meat Products: Health Aspects*.

[100] Zeng X., Xia W., Wang J. (2014). Technological properties of *Lactobacillus plantarum* strains isolated from Chinese traditional low salt fermented whole fish. *Food Control*.

[101] Nie X., Zhang Q., Lin S. (2014). Biogenic amine accumulation in silver carp sausage inoculated with *Lactobacillus plantarum* plus *Saccharomyces cerevisiae*. *Food Chemistry*.

[102] Quigley L., O'Sullivan O., Stanton C. (2013). The complex microbiota of raw milk. *FEMS Microbiology Reviews*.

[103] Ołdak A., Zielińska D., Rzepkowska A., Kołozyn-Krajewska D. (2017). Comparison of antibacterial activity of *Lactobacillus plantarum* strains isolated from two different kinds of regional cheeses from Poland: oscypek and korycinski cheese. *BioMed Research International*.

[104] Pisano M. B., Viale S., Conti S. (2014). Preliminary evaluation of probiotic properties of *Lactobacillus* strains isolated from Sardinian dairy products. *BioMed Research International*.

[105] Hulak N., Maksimović A. Ž., Kaić A., Skelin A., Fuka M. M. (2016). Indigenous strains of lactobacillus isolated from the istrian cheese as potential starter cultures. *Mljekarstvo*.

[106] Potočnjak M., Pušić P., Frece J., Abram M., Jankovic T., Gobin I. (2017). Three New *Lactobacillus plantarum* Strains in the Probiotic Toolbox against Gut Pathogen *Salmonella enterica* SerotypeTyphimurium. *Food Technology and Biotechnology*.

[107] Turchi B., Pedonese F., Torracca B. (2017). *Lactobacillus plantarum* and *Streptococcus thermophilus* as starter cultures for a donkey milk fermented beverage. *International Journal of Food Microbiology*.

[108] Patrovský M., Kouřimská L., Havlíková Š., Marková J., Pechar R., Rada V. (2016). Utilization of bacteriocin-producing bacteria in dairy products. *Mljekarstvo*.

[109] Ray R. C., Sharma P., Panda S. H. (2009). Lactic acid production from cassava fibrous residue using *Lactobacillus plantarum* MTCC 1407. *Journal of Environmental Biology*.

[110] Catte M., Gancel F., Dzierszinski F., Tailliez R. (1999). Effects of water activity, NaCl and smoke concentrations on the growth of *Lactobacillus plantarum* ATCC 12315. *International Journal of Food Microbiology*.

[111] Panda S. K., Ray R. C., Sharma H. K. (2016). Fermented foods and beverages from tropical roots and tubers. *Tropical Tuber crops: Technological Interventions*.

[112] Chen Y., Huang Y., Bai Y. (2017). Effects of mixed cultures of *Saccharomyces cerevisiae* and *Lactobacillus plantarum* in alcoholic fermentation on the physicochemical and sensory properties of citrus vinegar. *LWT-Food Science and Technology*.

[113] Liu J., Li L., Li B. (2017). Study on spoilage capability and VBNC state formation and recovery of *Lactobacillus plantarum*. *Microbial Pathogenesis*.

[114] Bravo-Ferrada B. M., Brizuela N., Gerbino E., Gómez-Zavaglia A., Semorile L., Tymczyszyn E. E. (2015). Effect of sucrose, trehalose and glutamate on the resistance to ethanol of frozen and freeze-dried acclimated oenological *Lactobacillus plantarum* strains. *Cryobiology*.

[115] Hashemi S. M. B., Khaneghah A. M., Barba F. J., Nemati Z., Sohrabi Shokofti S., Alizadeh F. (2017). Fermented sweet lemon juice (Citrus limetta) using *Lactobacillus plantarum* LS5: Chemical composition, antioxidant and antibacterial activities. *Journal of Functional Foods*.

[116] Hashemi S. M. B., Khaneghah A. M., Kontominas M. G. (2017). Fermentation of sarshir (kaymak) by lactic acid bacteria: antibacterial activity, antioxidant properties, lipid and protein oxidation and fatty acid profile. *Journal of the Science of Food and Agriculture*.

[117] Kim S., Kim J., Yun E. J., Kim K. H. (2016). Food metabolomics: From farm to human. *Current Opinion in Biotechnology*.

[118] Adebo O. A., Njobeh P. B., Adebiyi J. A., Gbashi S., Kayitesi E. (2017). Food metabolomics: a new frontier in food analysis and its application to understanding fermented foods. *Functional Food-Improve Health through Adequate Food*.

[119] Chen Y. W., Clore G. M. (2000). A systematic case study on using NMR models for molecular replacement: p53 tetramerization domain revisited. *Acta Crystallographica. Section D, Biological Crystallography*.

[120] Amapu T. Y., Ameh J. B., Ado S. A., Abdullahi I. O., Dapiya H. S. (2016). Amylolytic Potential of Lactic Acid Bacteria Isolated from Wet Milled Cereals, Cassava Flour and Fruits. *British Microbiology Research Journal*.

[121] Kanpiengjai A., Nguyen T.-H., Haltrich D., Khanongnuch C. (2017). Expression and comparative characterization of complete and C-terminally truncated forms of saccharifying *α*-amylase from *Lactobacillus plantarum* S21. *International Journal of Biological Macromolecules*.

[122] Panda S. H., Ray R. C. (2008). Direct conversion of raw starch to lactic acid by *Lactobacillus plantarum* MTCC 1407 in semi-solid fermentation using sweet potato (*Ipomoea batatas* L.) flour. *Journal of Scientific and Industrial Research*.

[123] Uppada S. R., Akula M., Bhattacharya A., Dutta J. R. (2017). Immobilized lipase from *Lactobacillus plantarum* in meat degradation and synthesis of flavor esters. *Journal of Genetic Engineering and Biotechnology*.

[124] Andersen H. J., Østdal H., Blom H. (1995). Partial purification and characterisation of a lipase from *Lactobacillus plantarum* MF32. *Food Chemistry*.

[125] An Y., Wang Y., Liang X. (2017). Purification and partial characterization of M1-UVs300, a novel bacteriocin produced by *Lactobacillus plantarum* isolated from fermented sausage. *Food Control*.

[126] Lei V., Amoa-Awua W. K. A., Brimer L. (1999). Degradation of cyanogenic glycosides by *Lactobacillus plantarum* strains from spontaneous cassava fermentation and other microorganisms. *International Journal of Food Microbiology*.

[127] Gouripur G., Kaliwal B. (2017). Screening and optimization of *β*-glucosidase producing newly isolated *Lactobacillus plantarum* strain LSP-24 from colostrum milk. *Biocatalysis and Agricultural Biotechnology*.

[128] Yevenes A., Frey P. A. (2008). Cloning, expression, purification, cofactor requirements, and steady state kinetics of phosphoketolase-2 from *Lactobacillus plantarum*. *Bioorganic Chemistry*.

[129] Vastano V., Capri U., Muscariello L., Marasco R., Sacco M. (2010). *Lactobacillus plantarum* adhesion and colonization: identification of adhesins and effects of intestinal environment on biofilm development. *Journal of Biotechnology*.

[130] Krishnan S., Gowda L. R., Karanth N. G. (2000). Studies on lactate dehydrogenase of *Lactobacillus plantarum* spp. involved in lactic acid biosynthesis using permeabilized cells. *Process Biochemistry*.

[131] Swain M. R., Ray R. C., Paramethioites S. (2016). Nutritional values and bioactive compounds in fermented fruits and vegetables. *Lactic Acid Fermentation of Fruits and Vegetables*.

[132] Li P., Gu Q., Yang L., Yu Y., Wang Y. (2017). Characterization of extracellular vitamin B 12 producing *Lactobacillus plantarum* strains and assessment of the probiotic potentials. *Food Chemistry*.

[133] Panda S. K., Ray R. C., Mishra S. S., Kayitesi E. (2017). Microbial processing of fruit and vegetable wastes into potential biocommodities: a review. *Critical Reviews in Biotechnology*.

[134] Arena M. P., Fiocco D., Massa S., Capozzi V., Russo P. (2014). *Lactobacillus plantarum* as a strategy for an in situ production of vitamin B2. *Journal of Food and Nutritional Disorders*.

[135] Lee K. W., Shim J. M., Park S.-K. (2016). Isolation of lactic acid bacteria with probiotic potentials from *kimchi*, traditional Korean fermented vegetable. *LWT- Food Science and Technology*.

[136] Gómez N., García D., Álvarez I., Raso J., Condón S. (2005). A model describing the kinetics of inactivation of *Lactobacillus plantarum* in a buffer system of different pH and in orange and apple juice. *Journal of Food Engineering*.

[137] Berbegal C., Peña N., Russo P. (2016). Technological properties of *Lactobacillus plantarum* strains isolated from grape must fermentation. *Food Microbiology*.

[138] Sakamoto K., Konings W. N. (2003). Beer spoilage bacteria and hop resistance. *International Journal of Food Microbiology*.

[139] Guimarães A., Santiago A., Teixeira J. A., Venâncio A., Abrunhosa L. (2017). Anti-aflatoxigenic effect of organic acids produced by *Lactobacillus plantarum*. *International Journal of Food Microbiology*.

[140] Zhou K., Zeng Y., Yang M. (2016). Production, purification and structural study of an exopolysaccharide from *Lactobacillus plantarum* BC-25. *Carbohydrate Polymers*.

[141] Gangoiti M. V., Puertas A. I., Hamet M. F. (2017). *Lactobacillus plantarum* CIDCA 8327: An *α*-glucan producing-strain isolated from kefir grains. *Carbohydrate Polymers*.

[142] Wang X., Shao C., Liu L., Guo X., Xu Y., Lü X. (2017). Optimization, partial characterization and antioxidant activity of an exopolysaccharide from *Lactobacillus plantarum* KX041. *International Journal of Biological Macromolecules*.

[143] Zhang L., Liu C., Li D. (2013). Antioxidant activity of an exopolysaccharide isolated from *Lactobacillus plantarum* C88. *International Journal of Biological Macromolecules*.

[144] Wang Y., Li C., Liu P., Ahmed Z., Xiao P., Bai X. (2010). Physical characterization of exopolysaccharide produced by *Lactobacillus plantarum* KF5 isolated from Tibet Kefir. *Carbohydrate Polymers*.

[145] Hidalgo-Cantabrana C., López P., Gueimonde M. (2012). Immune modulation capability of exopolysaccharides synthesised by lactic acid bacteria and bifidobacteria. *Probiotics and Antimicrobial Proteins*.

[146] Bakhshi N., Soleimanian-Zad S., Sheikh-Zeinoddin M. (2017). Dynamic surface tension measurement for the screening of biosurfactants produced by *Lactobacillus plantarum* subsp. *plantarum* PTCC 1896. *Enzyme and Microbial Technology*.

[147] Knockaert D., Raes K., Struijs K., Wille C., Van Camp J. (2014). Influence of microbial conversion and change in pH on iron-gallic acid complexation during lactobacillus fermentation. *LWT- Food Science and Technology*.

[148] Probst M., Fritschi A., Wagner A., Insam H. (2013). Biowaste: A Lactobacillus habitat and lactic acid fermentation substrate. *Bioresource Technology*.

[149] Bernbom N., Licht T. R., Saadbye P., Vogensen F. K., Nørrung B. (2006). *Lactobacillus plantarum* inhibits growth of *Listeria monocytogenes* in an in vitro continuous flow gut model, but promotes invasion of *L. monocytogenes* in the gut of gnotobiotic rats. *International Journal of Food Microbiology*.

[150] Messi P., Bondi M., Sabia C., Battini R., Manicardi G. (2001). Detection and preliminary characterization of a bacteriocin (plantaricin 35d) produced by a *Lactobacillus plantarum* strain. *International Journal of Food Microbiology*.

[151] Engelhardt T., Szakmár K., Kiskó G., Mohácsi-Farkas C., Reichart O. (2018). Combined effect of NaCl and low temperature on antilisterial bacteriocin production of *Lactobacillus plantarum* ST202Ch. *LWT-Food Science and Technology*.

[152] Shi Y., Zhai Q., Li D. (2017). Restoration of cefixime-induced gut microbiota changes by *Lactobacillus* cocktails and fructooligosaccharides in a mouse model. *Microbiological Research*.

[153] Khalil R., Mahrous H., El-Halafawy K., Kamaly K., Frank J., El Soda M. (2007). Evaluation of the probiotic potential of lactic acid bacteria isolated from faeces of breast-fed infants in Egypt. *African Journal of Biotechnology*.

[154] Temsahy M. M. E., Ibrahim I. R., Mossallam S. F., Mahrous H., Bary A. A., Salam S. A. A. (2015). Evaluation of newly isolated probiotics in the protection against experimental intestinal trichinellosis. *Veterinary Parasitology*.

[155] Hill C., Guarner F., Reid G. (2014). Expert consensus document: The International Scientific Association for Probiotics and Prebiotics consensus statement on the scope and appropriate use of the term probiotic. *Nature Reviews Gastroenterology & Hepatology*.

[156] Tong Y., Zhai Q., Lu W. New insights in integrated response mechanism of *Lactobacillus plantarum* under excessive manganese stress. *Food Research International*.

[157] Coghetto C. C., Brinques G. B., Siqueira N. M., Pletsch J., Soares R. M. D., Ayub M. A. Z. (2016). Electrospraying microencapsulation of *Lactobacillus plantarum* enhances cell viability under refrigeration storage and simulated gastric and intestinal fluids. *Journal of Functional Foods*.

[158] Wang Z., Wu Q., Kuča K., Dohnal V., Tian Z. (2014). Deoxynivalenol: Signaling pathways and human exposure risk assessment—an update. *Archives of Toxicology*.

[159] Gupta R., Srivastava S. (2014). Antifungal effect of antimicrobial peptides (AMPs LR14) derived from *Lactobacillus plantarum* strain LR/14 and their applications in prevention of grain spoilage. *Food Microbiology*.

[160] Kaushik J. K., Kumar A., Duary R. K., Mohanty A. K., Grover S., Batish V. K. (2009). Functional and probiotic attributes of an indigenous isolate of *Lactobacillus plantarum*. *PLoS ONE*.

[161] Tang W., Xing Z., Li C., Wang J., Wang Y. (2017). Molecular mechanisms and in vitro antioxidant effects of *Lactobacillus plantarum* MA2. *Food Chemistry*.

[162] Thirabunyanon M., Hongwittayakorn P. (2013). Potential probiotic lactic acid bacteria of human origin induce antiproliferation of colon cancer cells via synergic actions in adhesion to cancer cells and short-chain fatty acid bioproduction. *Applied Biochemistry and Biotechnology*.

[163] Dowarah R., Verma A. K., Agarwal N. (2017). The use of *Lactobacillus* as an alternative of antibiotic growth promoters in pigs: a review. *Animal Nutrition*.

[164] Nayak S. K. (2010). Probiotics and immunity: a fish perspective. *Fish & Shellfish Immunology*.

[165] Akhter N., Wu B., Memon A. M., Mohsin M. (2015). Probiotics and prebiotics associated with aquaculture: a review. *Fish and Shellfish Immunology*.

[166] Minic R., Gavrovic-Jankulovic M., Petrusic V. (2015). Effects of orally applied Fes p1-displaying *L. plantarum* WCFS1 on Fes p1 induced allergy in mice. *Journal of Biotechnology*.

[167] Yu L., Zhai Q., Tian F. (2017). *Lactobacillus plantarum* CCFM639 can prevent aluminium-induced neural injuries and abnormal behaviour in mice. *Journal of Functional Foods*.

[168] Kwon E. K., Kang G.-D., Kim W.-K., Han M. J., Kim D.-H. (2017). *Lactobacillus plantarum* LC27 and *Bifidobacterium longum* LC67 simultaneously alleviate ethanol-induced gastritis and hepatic injury in mice. *Journal of Functional Foods*.

[169] Arasu M. V., Al-Dhabi N. A., Ilavenil S., Choi K. C., Srigopalram S. (2016). In vitro importance of probiotic *Lactobacillus plantarum* related to medical field. *Saudi Journal of Biological Sciences*.

[170] Murofushi Y., Villena J., Morie K. (2015). The toll-like receptor family protein RP105/MD1 complex is involved in the immunoregulatory effect of exopolysaccharides from *Lactobacillus plantarum* N14. *Molecular Immunology*.

[171] Ducrotté P., Sawant P., Jayanthi V. (2012). Clinical trial: *Lactobacillus plantarum* 299v (DSM 9843) improves symptoms of irritable bowel syndrome. *World Journal of Gastroenterology: WJG*.

[172] Mihailović M., Živković M., Jovanović J. A. (2017). Oral administration of probiotic *Lactobacillus paraplantarum* BGCG11 attenuates diabetes-induced liver and kidney damage in rats. *Journal of Functional Foods*.

